# Incidence of de novo HPV infections in a previous HPV-negative group, related to use of different contraceptive methods: a retrospective cohort study

**DOI:** 10.1186/s13027-025-00688-6

**Published:** 2025-08-05

**Authors:** Lina Jans, Jan Brynhildsen, Joar Hofgaard, Safia Ansari, Lovisa Eklöf, Lovisa Bergengren

**Affiliations:** 1 Department of Women’s Health, Region Örebro County, Örebro, 701 16 Sweden; 2 School of Medical Sciences, Faculty of Medicine and Health, Örebro University, Örebro, Sweden

**Keywords:** Human papillomavirus, Uterine cervical dysplasia, Intrauterine device, Contraception

## Abstract

**Background:**

Users of intrauterine devices (IUDs) have been found to have a lower incidence of cervical cancer in meta-analyses, but these studies have not been able to examine the influence of IUD type. The aim of this study is to investigate the incidence of de novo high-risk human papillomavirus (HPV) infections in relation to the reported use of contraceptive methods, with special regard to different types of IUDs.

**Methods:**

A sample of participants in the national screening program for cervical cancer (*n* = 11,702) with a negative HPV test in 2017–2018 were included. Their subsequent HPV test results in 2020–2023 were analyzed in relation to their reported contraceptive method.

**Results:**

Participants who reported use of hormonal contraception had higher incidence of a positive HPV screening test (5.6%) compared with women with no reported contraception (4.2%) (OR 1.29; 95% CI 1.01–1.64). There was no significant difference in HPV incidence among women who reported use of hormonal IUD (HIUD) or copper-containing IUD (CU-IUD). Women who reported use of the same contraceptive method in both screening rounds showed no significant differences in HPV incidence, regardless of the contraceptive method they had used.

**Conclusion:**

The incidence of de novo HPV infections is not significantly different in users of different types of IUD.

## Background

Cervical cancer is the fourth most common malignancy among women worldwide. The incidence varies widely around the world, and nearly 90% of deaths due to cervical cancer occur in low- and middle-income countries [1]. The main etiologic agent in cervical cancer is persistent human papillomavirus (HPV) infection [2]. HPV infection is a common sexually transmitted disease [3, 4], and although usually transient, HPV infections, when persistent, may cause cervical dysplasia that can progress to cervical cancer if left untreated. HPV vaccination and screening to find premalignant lesions are important strategies for preventing cervical cancer. Access to these strategies is unevenly distributed in the world. More than 85% of high-income countries, but less than 25% of low-income countries, have implemented HPV vaccination in their national immunization programs [1]. Therefore, there is still a great need to better understand the risk factors as well as factors associated with a lower risk of cervical cancer.

Intrauterine devices (IUDs) are contraceptive methods widely used globally, with copper-containing IUDs (Cu-IUDs) and hormonal IUDs (HIUDs) as the most common types [5]. A meta-analysis published in 2017 showed that women who reported ever using an IUD had a lower reported incidence of cervical cancer (odds ratio (OR) 0.64; 95% CI 0.53–0.77). However, this meta-analysis was unable to examine the influence based on IUD type [6]. Data were collected 1981–2006, a time span when both Cu-IUDs and HIUDs, as well as inert IUDs, were in use.

Studies on cervical dysplasia and HPV in IUD users have reported conflicting results. A retrospective cohort analysis found a lower relative risk of high-grade cervical dysplasia in Cu-IUD users compared to HIUD users [7], while another cohort study using register data found no difference in risk between the two groups, although both had a lower risk than women using oral contraceptives (OC) [8].

A retrospective analysis by Lekovich found that insertion of Cu-IUD resulted in a higher clearance rate of HPV than insertion of HIUD in a group of 302 women. However, no control group was included, and no difference in effect depending on IUD type on cervical dysplasia was detected [9]. So far, no large study has prospectively examined the risk of HPV infection in relation to use of different types of IUDs. Since access to HPV vaccination and cervical cancer screening programs varies around the world, it is important to examine whether use of Cu-IUD can potentially lower the risk of HPV infections and thereby be a cheap option to prevent cervical cancer in women living in countries with limited access to HPV vaccination and screening programs.

The screening program for cervical cancer in Sweden is based on primary HPV testing, with cytology as a triage, and was implemented in Region Örebro County in September 2016 for women above the age of 30 years. Women participating in the program were invited every third year, but later a prolonged interval has been common due to the COVID-19 pandemic. Since 2023 the screening interval for HPV-negative women has been extended to every fifth year for women aged 23 to 49 years, and every seventh year for women aged 50–70 years.

Our previous research on HPV prevalence found that use of HIUD was associated with significantly higher odds of a positive HPV screening test when compared to those of women not using hormonal contraception or IUD. For women using Cu-IUD, no significant difference was found [10]. However, no information on previous screening tests was available, and therefore, it is unknown whether the HPV infections were persistent or transient.

In this study the aim is to examine the incidence of de novo HPV infections in a population of women with a previous negative HPV test, in relation to reported use of contraceptive method, with special regard to different types of IUDs.

## Materials and methods

### Study design

This is a retrospective cohort study analyzing results from women participating in the screening program for cervical cancer in Region Örebro County, Sweden, in 2017–2023.

### Study setting

In Region Örebro County, Sweden, the HPV tests in the screening program for cervical cancer are performed by midwifes in primary care settings. The samples are analyzed at the accredited laboratory of clinical pathology, Örebro University hospital (SWEDAC no 1351), using an mRNA-based assay, Aptima (Hologic, Marlborough, MA, USA). All HPV-positive samples are analyzed for cytological abnormalities according to national guidelines [11]. Cytological assessment is performed by IAC-certified cytotechnicians and classified as normal cytology, atypical squamous cells of undetermined significance (ASCUS), low-grade squamous intraepithelial lesion (LSIL), high-grade squamous intraepithelial lesion (HSIL), atypical glandular cells, unclear atypia, or suspicion of cancer, according to the international Bethesda classification [12].

At the time of the sampling, the participating women fill in a record with questions regarding contraceptive use, menopausal status, menopausal hormonal treatment, ongoing pregnancy and delivery the previous year. Women tick the boxes for those matters that apply and leave blank those that do not apply. Contraceptive use is divided into boxes for hormonal contraception, including both combined hormonal contraception and progestin-only methods, and intrauterine devices, divided into Cu-IUD and HIUD.

### Study participants

All women aged 30–49 years who participated in the screening program in Region Örebro County in 2017–2018 were included in a previous study that examined the prevalence of HPV and dysplasia among IUD users [10]. From this cohort all women who had a negative HPV test in 2017–2018 and a new HPV test in the screening program in Region Örebro County during the years 2020–2023 were included in this study. Consequently, all HPV positive women in the screening round 2020–2023 were regarded as “de novo” HPV infections since all included women had a negative HPV test in the previous screening round.

No power analysis was performed, since this was a follow-up from a previous study.

Women who reported ongoing pregnancy at the screening occasion in 2020–2023 were excluded. Based on the reported information from the screening records, the cohort was categorized as follows: users of hormonal contraception, HIUD, Cu-IUD, and no reported contraception. Women who ticked boxes for both hormonal contraception and HIUD were regarded as HIUD users in the analysis. Data for those who reported use of both hormonal contraception and Cu-IUD were regarded as missing data. When all the boxes for contraception were left blank, the women were regarded as having “no reported contraception.”

The information from the screening records from both the initial screening test in 2017–2018 and from the next screening round in 2020–2023 was paired with the latter HPV test result from the screening round in 2020–2023. A separate analysis was performed on the results from the women who reported the same contraceptive use in both screening rounds, in this article referred to as “long-term users.”

No informed consent was needed, since the included women had already been informed about their test results and followed-up according to national guidelines.

### Statistical analysis

All statistical analyses were performed in SPSS version 29 (IBM SPSS, New York, NY, USA). Logistic regression analyses were used to estimate odds ratios and 95% confidence intervals (95% CIs).

### Ethical approval

The study was approved by the Swedish Ethical Review Authority (Dnr 2021 − 01379, 2022-01091-02, 2023-03601-02 and 2024-05319-02).

## Results

**Fig. 1:**
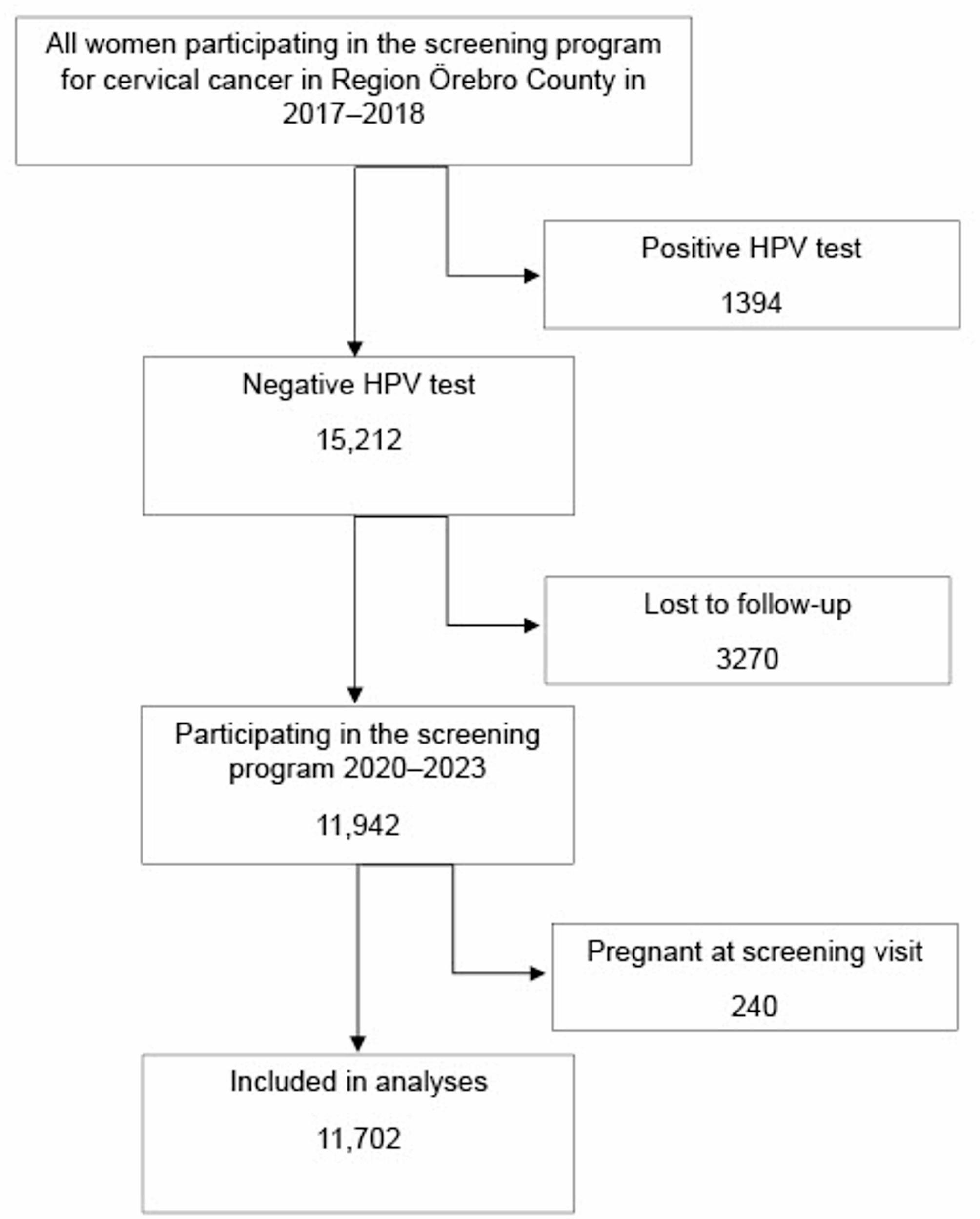
Flow chart of inclusion, exclusion, and missing data

A total of 78.5% of the participants with a negative HPV test in the screening program in the years 2017–2018 had the next HPV test taken in the screening program in Region Örebro County in 2020–2023. After excluding 240 women who were pregnant at the time of the next screening, 11,702 women were included in the analysis (Fig. 1). Of these women, 523 (4.5%) had a positive HPV test in 2020–2023.

The mean age at the first screening test in 2017–2018 was 39.4 years (30–49). Of the participating women in 2020–2023, 1504 (12.9%) were postmenopausal and 322 (2.8%) reported menopausal treatment. The most common type of contraception reported by the women was HIUD (2780 women, 23.8%), whereas 685 women (5.9%) reported Cu-IUD. Hormonal contraception was reported by 1635 women (14.0%) (Table 1). Data on contraception were missing in 18 cases.

**Table 1:** Reported data in the screening records and HPV incidence in the different categories of participants

	No. of women (%)	No. of HPV-positive women	HPV incidence
**Total**	**11**,**702**	**523**	**4.5%**
Hormonal contraception	1635 (14.0%)	91	5.6%
Hormonal IUD	2780 (23.8%)	126	4.5%
Copper-IUD	685 (5.9%)	26	3.8%
No reported contraception	6584 (56.3%)	279	4.2%
Postmenopausal	1504 (12.9%)	68	4.5%
Menopausal hormonal treatment	322 (2.8%)	13	4.0%
Within one year postpartum	363 (3.1%)	14	3.9%

Women who reported use of hormonal contraception had higher incidence of a positive HPV screening test compared with women with no reported contraception (5.6% vs. 4.2%, aOR 1.29; 95% CI 1.01–1.64). There was no significant difference in HPV incidence among women who reported use of HIUD or Cu-IUD (Table 2). Neither was there a significant difference in HPV incidence when comparing HIUD and Cu-IUD users (aOR 1.25; 95% CI 0.81–1.93).

**Table 2:** Odds of having an HPV-positive test in relation to reported use of contraception

Reported contraception	OR (95% CI)	aOR^a^ (95% CI)
No reported contraception	Ref	Ref
Hormonal contraception	1.33 (1.05–1.70)	1.29 (1.01–1.64)
Hormonal IUD	1.07 (0.87–1.33)	1.09 (0.88–1.35)
Copper-IUD	0.89 (0.59–1.34)	0.87 (0.58–1.31)

^a^Adjusted for age

75% of the participating women reported use of the same contraceptive method in both screening rounds and were thus classified as long-term users. There were no significant differences in HPV incidence in long-term users of different types of contraception (Table 3).

**Table 3:** Odds of having an HPV-positive test among long-term users of different types of contraception

Reported contraception	No^a^ (% of total)	OR (95% CI)	aOR^b^ (95% CI)
No reported contraception	5421 (82%)	Ref	Ref
Hormonal contraception	1069 (65%)	1.26 (0.92–1.73)	1.23 (0.90–1.68)
Hormonal IUD	1809 (65%)	1.21 (0.93–1.57)	1.28 (0.99–1.67)
Copper-IUD	473 (69%)	0.94 (0.57–1.55)	0.93 (0.56–1.54)

^a^ No of long-term users i.e., those women who reported the same use of contraception in the previous screening round

^b^ Adjusted for age

The vast majority of the HPV-positive women had normal cytology, ASCUS, or LSIL (Table 4).

**Table 4:** Cytology results among the 523 HPV positive women

Cytology results	No. of women	Percentage
Normal cytology	212	40.5%
ASCUS/LSIL	281	53.7%
HSIL	22	4.2%
Unclear atypia	5	1.0%
Not assessable	3	0.6%

## Discussion

No significant difference was found in the incidence of de novo HPV infections in women with a previous HPV-negative screening test reporting use of different types of IUD at the next screening round three to five years later. Long-term use of IUD did not affect the incidence. The participating women were followed for two screening rounds, providing a unique opportunity to study long-term users of both HIUD and Cu-IUD.

Women with reported use of hormonal contraception had a higher risk of being HPV-positive, which is in accordance with results from numerous previous studies. A meta-analysis by the International Collaboration of Epidemiological Studies of Cervical Cancer in 2007 found a higher risk for cervical cancer in women using hormonal contraception [13], and the International Agency for Research on Cancer has classified combined oral contraceptives as a cause of cervical cancer [14]. But a review by Anastasiou found no increased risk for cervical dysplasia or cancer after controlling for HPV infection [15].

In the screening records it is not specified what type of hormonal contraception the women are using when reporting use of hormonal contraception. Both users of combined contraceptives (pills, patches, rings) and progestin-only methods (pills, injectables, implants) are included in this group, and consequently, it is difficult to draw conclusions regarding type of hormonal contraception in relation to the higher incidence of HPV infections in this group of women.

Women not reporting use of contraception were chosen as reference in the statistical analyses, to avoid hormonal contraception being a bias. The non-user group also includes women using condom as contraception, and women who are not sexually active, and therefore may constitute a low-risk group for acquiring HPV infections. We cannot rule out that this may have biased the results, which therefore must be interpreted with caution. On the other hand, this group may also include women who engage in unprotected sex, thus carrying a higher risk of acquiring an HPV infection.

Other confounding factors for acquiring HPV infection, such as number of sexual partners, smoking, HPV vaccination etc., are not noted in the screening records and therefore not analyzed in this study, which is a weakness. The earlier studies on cervical cancer incidence in IUD users have, in varying degree, taken some of these factors into account, and still found a lower cervical cancer incidence in IUD users [6]. It is not known if these confounding factors are different among users of different kinds of IUD. HPV vaccination was introduced in the Swedish national vaccination program in 2010 and since then it is offered to all girls in the age of 10–12 years. The women in the age-groups included in the present study have not been offered HPV vaccination in the regular vaccination program. The rate of HPV vaccination in these age-groups and if there is a difference in vaccination status between groups, is unknown.

Roughly 20% of the women aged 30–49 years who participated in the screening program in 2017–2018 have not participated in the program in Region Örebro County since then. Some women may have moved out of the county and undergone their next screening test in a different region. We consider the risk that this may have biased our results as small. A power analysis was undertaken for the original study [10], but no power analysis was undertaken for this secondary analysis of the material. Most likely the present study includes a low-risk group and may be underpowered, and the number of participants too small to detect small but significant differences between users of different types of contraception. Moreover, the number of Cu-IUD users in the cohort was smaller than expected in the power analysis. However, it can be noted that the odds ratios for Cu-IUD users were generally lower than for users of hormonal IUDs and hormonal contraception, although the differences are not significant. This may warrant further studies with larger sample sizes.

Screening programs for cervical cancer have been based on cytology, but HPV-based screening programs have proven to be more effective and been implemented throughout the world [16]. Region Örebro County was one of the early adopters of an HPV-based screening program, providing a unique opportunity to investigate the relationship between HPV incidence and contraceptive methods. However, the number of women who have participated in the HPV-based screening program may still be too limited to draw definitive conclusions.

The study [10] that provides the basis for this study included women aged 30–49 years. This age group was selected because women in this range were the first to be screened with a primary HPV test and also most likely represented a group in need of contraception. In this follow-up study, the women were now three to five years older, and thus some of them were menopausal and no longer in need of contraception. Still, a majority of the women using an IUD in 2017–2018 were using the same type of IUD in the follow-up. A weakness of this study is that we cannot know whether the women were using the same contraception during the years between the screening opportunities; only active use at the time of the screening tests is reported by the participating women. However, it seems likely that women who reported use of an IUD in 2017–2018 and reported use of the same contraceptive method three to five years later actually had used an IUD during this time span.

The mechanism behind the lower incidence of cervical cancer among IUD users, reported by Cortessis et al., is unclear. It has been speculated that copper from the Cu-IUD creates an inflammatory response that clears HPV infections. HIUD, on the other hand, has an anti-inflammatory response that promotes persistence of HPV infections [9]. Since we found no difference in the HPV incidence in either users of Cu-IUD or users of HIUD compared to women not using contraception, there might be reasons other than differences in HPV incidence that explain the lower incidence of cervical cancer. Copper complexes induce aggregation of the HPV oncoprotein E6, and thereby stabilize p53, which has tumor-suppressive functions [17, 18]. However, it remains speculative whether this occurs in cervical cells, as no studies have yet examined whether the copper from the Cu-IUD is absorbed by the cervix in Cu-IUD users. If that is the case, it would be valuable to explore whether Cu-IUD use can help prevent development of cervical dysplasia in HPV-infected women.

The studies on prevalence of cervical dysplasia among IUD users show conflicting results [7, 8], highlighting the need for further research on how IUDs may affect the progression from HPV infection to cervical cancer, and whether there is a difference between HIUD and Cu-IUD.

## Conclusions

Incidence of de novo HPV infections are not significantly different in users of different types of IUDs.

## Data Availability

The dataset used and analyzed during the current study are available from the corresponding author on reasonable request.

## Abbreviations

IUD: Intrauterine device
HPV: High-risk human papillomavirus
HIUD: Hormonal IUD
CU: IUD-copper-containing IUD
OC: Oral contraceptives
ASCUS: Atypical squamous cells of undetermined significance
LSIL: Low-grade squamous intraepithelial lesion
HSIL: High-grade squamous intraepithelial lesion
CI: Confidence interval
OR: Odds ratio
